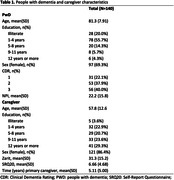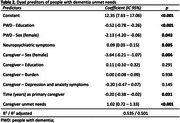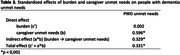# More than burden: the relationship between caregivers and people with dementia unmet needs

**DOI:** 10.1002/alz.089211

**Published:** 2025-01-09

**Authors:** Laiss Bertola, Fabiana A F da Mata, Ari Alex Ramos, Carolina Godoy, Haliton Alves de Oliveira Júnior, Cleusa P Ferri

**Affiliations:** ^1^ Hospital Alemão Oswaldo Cruz, São Paulo, São Paulo Brazil; ^2^ Universidade Federal de São Paulo (UNIFESP), São Paulo, São Paulo Brazil; ^3^ Universidade Federal de São Paulo, São Paulo Brazil; ^4^ Universidade Federal de São Paulo (UNIFESP), São Paulo, São Paulo/SP Brazil

## Abstract

**Background:**

People with dementia (PWD) have several unmet needs during the syndrome progression. More unmet needs are related to worse outcomes, such as hospitalizations, injuries, and death. The caregiving burden has been related to more PWD unmet needs. However, looking beyond the burden to a broader impact of caring activities on the carer’s life is necessary. This study aims to verify if caregivers' unmet needs predict PWD unmet needs beyond the burden.

**Method:**

In‐home interviews of a nationally distributed sample of 140 dyads (caregiver and PWD) assessed the care needs of both using the Johns Hopkins Dementia Care Needs Assessment (JHDCNA 2.0). We considered each item as 0 ‐ not needed or needed and fully met, or 1 – needed and partially met or unmet. Beyond the care assessment, the interview included several measures, including sociodemographic, caregiver burden (Zarit Burden Scale), mental health, quality of life, and information about dementia care. We conducted regression models to verify which information of the dyad best predicted more PWD unmet needs. Additionally, we performed a mediation analysis to confirm if the caregiver’s burden impacts the PWD unmet needs by its effects on the caregiver’s unmet needs.

**Result:**

PWD had at least two unmet needs of 44 (M = 18,1; SD = 6,9), the caregiver had a mean of 11,4 (SD = 3,8) unmet needs, and 71.4% reported burden (Table 1). The PWD being male, having less education, and having more neuropsychiatric symptoms, combined with the caregiver being male, having fewer years as the primary caregiver, and having more unmet needs were associated with more PWD unmet needs (Table 2). Mediation results indicate that the burden only indirectly affects the PWD’s unmet needs through the unmet needs of the caregiver (Table 3).

**Conclusion:**

Unmet needs are a reality for the dyad. Considering that family members predominantly provide care in low‐ and middle‐income countries, supporting caregivers is essential for their health and for them to be able to reduce the unmet needs of PWD. The impact of the caregiving burden extends beyond mental health, operating in a complex environment of unmet needs.